# Effects of Lower Limb Proprioceptive Training on Balance and Trunk Control Among the Adult Stroke Population

**DOI:** 10.7759/cureus.64554

**Published:** 2024-07-15

**Authors:** Shobhna Mishra, Ankit Jain, Prateek Sharma, Ghazala Khan, Bhumika Chhibber

**Affiliations:** 1 Department of Physiotherapy, Amity Institute of Health Allied Sciences, New Delhi, IND; 2 Department of Physiotherapy, Indian Head Injury Foundation, New Delhi, IND; 3 Department of Physiotherapy, Banarsidas Chandiwala Institute of Physiotherapy, New Delhi, IND; 4 Department of Rehabilitation, Indian Spinal Injuries Centre, New Delhi, IND

**Keywords:** exercises, trunk control, stroke, proprioception, balance

## Abstract

Background and objective

Balance and trunk control are major concerns among older adults during stroke rehabilitation. Loss of proprioception in the affected limb has a greater influence on motor learning and reeducation during balance training. Available studies stress the relevance of strength and functional training in regaining balance and trunk control. Proprioception training, in addition to available rehabilitation, can optimize the balance among this population. Our study aimed to find out the effects of proprioceptive training on balance and trunk control among the chronic stroke population.

Methodology

Out of 45 subjects enrolled at the Indian Head Injury Foundation, New Delhi, India, 30 subjects were selected based on selection criteria and randomized into two groups using the chit method, with 15 subjects in each group. The control group received conventional training, which included a range of motion, stretching, and strengthening exercises, while the intervention group received additional proprioceptive training five days per week for four consecutive weeks. Subjects were assessed on the Berg Balance Scale and Trunk Control Test for balance and trunk control on day one and after four weeks. A paired t-test was used to analyze the difference within the groups, and unpaired t-tests were used between the groups, keeping p < 0.05 as a significance level.

Results

After four weeks of intervention, statistically significant improvements were seen in the balance and trunk control groups, with p < 0.05 in both groups. However, a significant improvement in balance was observed in the experimental group when compared across groups (p = 0.001), whereas no statistically significant improvement in trunk control was found (p = 0.061).

Conclusion

We conclude that proprioceptive training and conventional physiotherapy both help to improve balance. Proprioceptive training is better for improving balance, but it has no significant effects on trunk control. It is likely that an extended intervention time or a different form of intervention may be required to achieve substantial gains in these areas. Future research might look at other outcome measures or the impacts of other types of therapies to see which ones are most helpful at increasing trunk control.

## Introduction

Stroke is classically characterized as a neurological deficit attributed to an acute focal injury of the CNS by a vascular cause [1], with high rates of morbidity, mortality, and impairment that place a significant financial strain on households and society [2,3]. Symptoms may last up to 24 hours, resulting in an incomplete recovery that may lead to severe disability. Stroke is the fourth most common cause of death and the fifth most common cause of disability in India [4]. The prevalence of stroke is markedly higher in men than in women in all age groups, except the age group between 20 and 39 years [5]. Generally, men are 30% more prone to having a stroke than women, but in intracerebral hemorrhage, there is very little gender difference [6]. The risk of having a stroke increases with increasing age in both men and women, especially in people whose age is more than 50 years [5]. The mortality rate after a stroke ranges from 13% to 35%. Countries with low and middle incomes have a higher fatality rate [7]. According to available data, economic hardship not only affects the risk factors and prevalence of stroke but also the severity and mortality of strokes, as well as the rate of stroke at lower ages [8].

Typically, stroke patients present with impaired senses, cognition, and proprioception, which greatly affect their balance, resulting in decreased motor skills and making the person physically dependent. It has been found that around 83% of the stroke population has difficulty maintaining balance [9]. Balance is defined as the ability to maintain equilibrium in the gravitational field by maintaining body mass over its base of support. Balance is a complicated process; the maintenance of the position is controlled by postural adjustments to voluntary activity and corresponds to external perturbation [10]. Balance impairment is characterized by short supporting time, disparities between two sides of the body, and sluggish walking pace, all of which may increase the risk of falling [11].

Trunk instability is also one of the problems in survivors following stroke; trunk control and sitting balance are thought to be important determinants of functional prognosis and hospital stay. Several studies have already looked into how trunk training affects trunk control, mobility, and balance while sitting and standing [12]. As stroke patients lose their capacity to perform postural adjustment and maintain postural alignment due to spasticity, weakness, loss of equilibrium, and righting reactions, the trunk assumes an asymmetrical posture. Since the trunk is the central pivot point of the body, proximal trunk control is a requirement for distal limb movement control, balance, and functional mobility [13]. Changes in trunk position perception and trunk muscular weakening in stroke patients have a major impact on balance. Anticipatory postural changes of trunk muscles play a significant role in sustaining antigravity postures such as sitting and standing while a reaching activity is performed [14].

Proprioception is the position sense of body parts in space; this is sensory information that is derived from the muscle spindle, Golgi tendon organ, and receptors present in the joint. For better movement control and function, proprioception is important [15]. A proprioceptive deficit may occur due to damage to the proprioceptive receptor; 54-64% of the population experience a proprioception deficit after stroke [16]. Several studies suggest that lower limb proprioception is more affected than the upper limb in post-stroke cases [17]. Impaired proprioception can lead to poor postural control, impaired gate, and increased fall risk, which also results in poor functional outcomes [18]. It not only leads to muscle weakness but also causes balance impairment [19]. For optimal balance control, the CNS integrates visual, vestibular, and proprioceptive information to produce motor commands that coordinate the activation patterns of muscles [20]. Proprioception plays a crucial role in balance control as it involves one’s ability to integrate the sensory signals from various mechanoreceptors to thereby determine body position and movement in space [21]. However, despite knowledge of proprioception's crucial role in balance control, few studies have focused on this factor in patients with chronic stroke.

According to Ryerson et al., proprioceptive exercise is necessary to enhance balance and trunk control [22]. However, there are very few existing studies on the effects of proprioceptive training on balance and trunk control in chronic stroke patients. In addition, the carryover effect of proprioceptive training on balance and trunk control in chronic stroke is insufficient. Also, most studies on proprioceptive training in chronic stroke patients are based on acute stroke patients, and studies on chronic stroke lack quantity. Therefore, this study is designed to investigate the effects of proprioceptive training on balance and trunk control in patients with chronic stroke.

## Materials and methods

**Figure 1:**
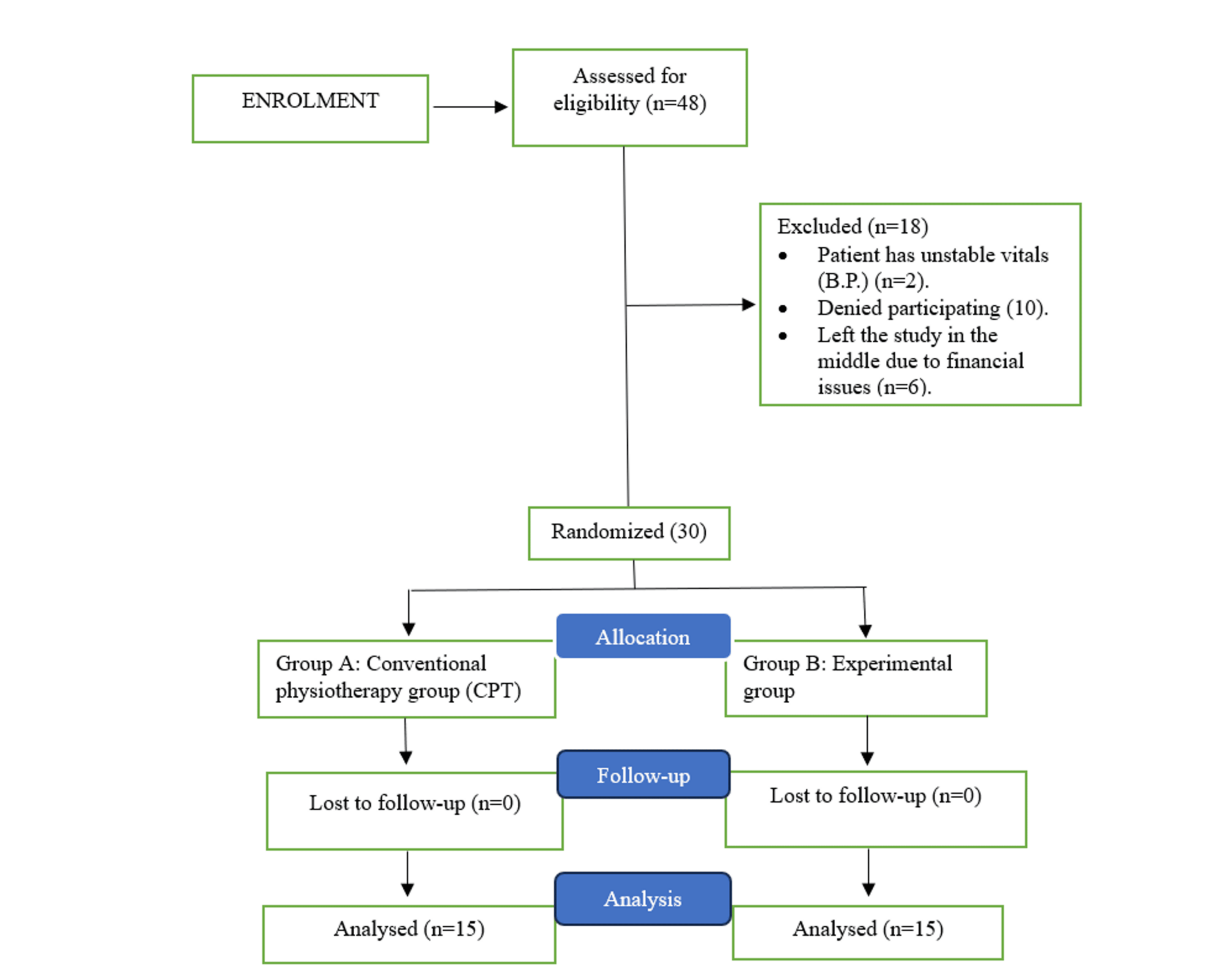
Study procedure

Out of 45, 30 stroke patients at the Indian Head Injury Foundation, New Delhi, India, were enrolled; informed consent was obtained from all enrolled participants before initiating the study protocol. Subjects were randomly assigned to two groups using the chit method. This study used a single-blinding method, which ensured that patients were unaware of their assigned group throughout the experiment. The intervention was applied to the experimental group (n = 15) and the conventional physiotherapy (CPT) group (n = 15). In this pre-test and post-test experimental design (comparative) study, detailed explanations and demonstrations of each test were performed for the participants before taking measurements. All data processing and statistical analysis were conducted with IBM SPSS Statistics for Windows, Version 19.0 (Released 2010; IBM Corp., Armonk, NY, USA) to compute means and SDs. All participants were tested for normality, and general characteristics were examined through descriptive statistics. We used a paired t-test to analyze changes in the variables pre- and post-treatment within each group. To compare differences in the variables between groups, an independent sample t-test was used. The statistical significance level for all data was set at p < 0.05. Figure 1 shows the flowchart of the study’s methodology.

Inclusion and exclusion criteria

The inclusion criteria for the study were as follows: unilateral hemiplegia with lower limb involvement; duration of having a stroke should be at least six months; ability to understand the directions from the researchers (those with a Montreal cognitive assessment (MOCA) score of 26 or greater); subjects of both genders were included between 35 and 55 years of age; and no history of other neurological conditions that can affect balance. The exclusion criteria were as follows: severely impaired balance; unstable overall health condition; pre-diagnosed visual or hearing impairment (even after correction); and those deemed unable to participate in the study by the researchers. The outcome variables are balance and trunk control. The tools used are the Berg Balance Scale (BBS) and the Trunk Control Test (TCT). This research was approved by the Amity Institute of Health Allied Sciences (AIHAS) review board at Amity University.

Intervention

**Figure 2:**
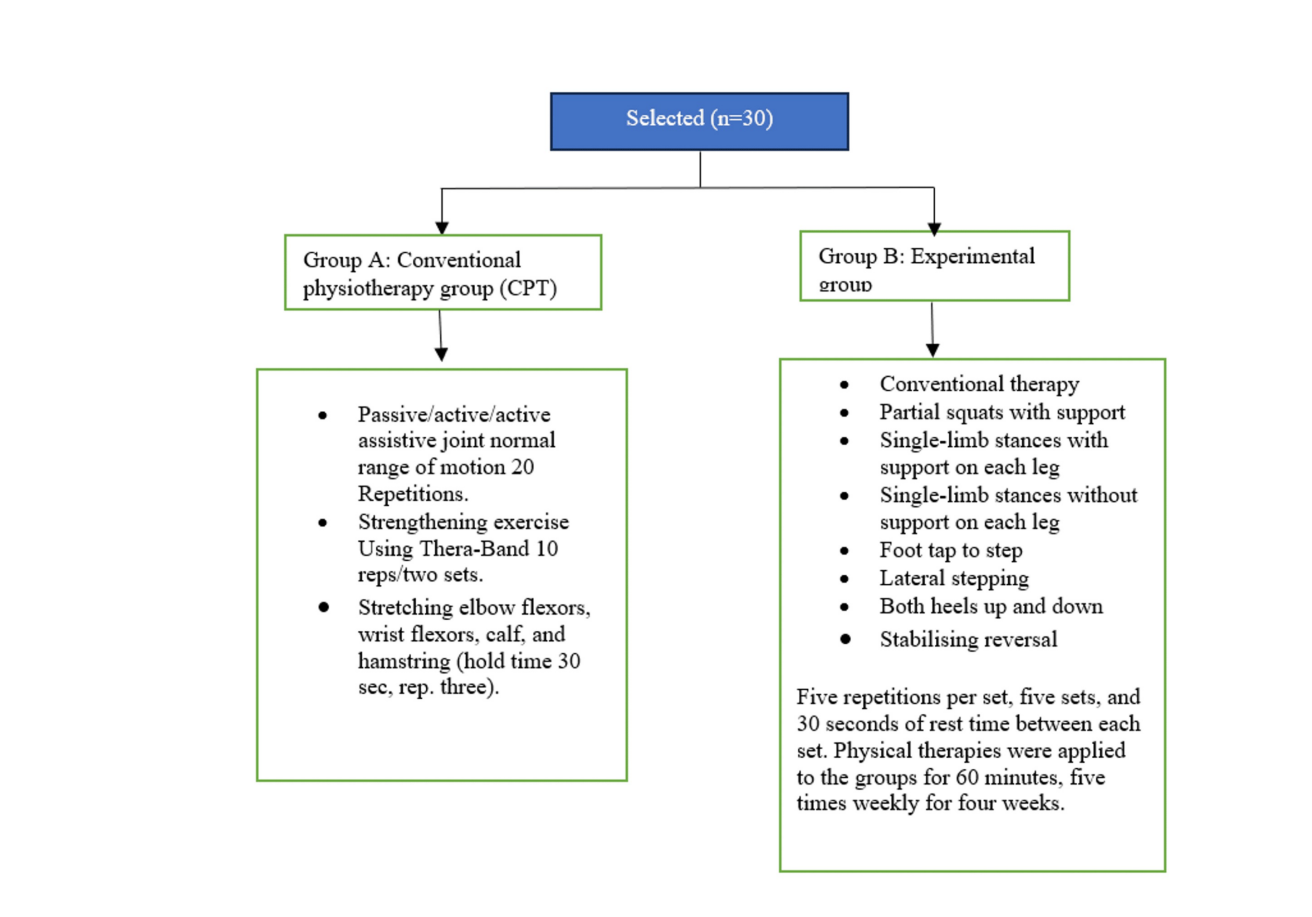
Interventions provided in both groups

The conventional therapy program used in the study (Group A) includes passive, active, and active assistive joint normal range of motion with 20 repetitions; strengthening exercises using Thera-Band with 10 reps in two sets; and stretching of elbow flexors, wrist flexors, calf, and hamstring with a 30-second hold time and three repetitions. The proprioceptive-based training (Group B) includes conventional therapy, partial squats with support, single-limb stances with and without support on each leg, foot tap to step, lateral stepping, both heels up and down, and stabilizing reversal with five repetitions per set, five sets, and 30 seconds of rest time between each set (Figure 2). Physical therapies were applied to the groups for 60 minutes, five times weekly, for four weeks.

## Results

The participant demographics in Table 1 show a comparison between the CPT group and the experiment group for age, height, weight, and BMI. There was no statistically significant difference (p > 0.05) between the experimental and CPT groups in age (p = 0.306, t = -5.7), height (p = 0.866, t = 0.351), weight (p = 0.941, t = 0.396), or BMI (p = 0.895, t = -0.769). There was no statistically significant difference between the experiment and the CPT group in gender (p = 0.715) or side (p = 0.068). However, there is a significant difference found in the type of stroke (ischemic and hemorrhagic), with p = 0.001. In the experimental group and CPT group, 53.3% of patients were female, and 46.7% of patients were male. The majority of 66.7% of patients had the left affected side, and 33.3% of patients had the right in the experimental group and CPT group, respectively. A total of 80% of patients had ischemic stroke, and 20% had hemorrhagic stroke in the experimental group and CPT group, respectively.

**Table 1:** Descriptive statistics of age, height, weight, and BMI among chronic stroke patients CPT, conventional physiotherapy

Variable	CPT group (mean ± SD)	Experimental group (mean ± SD)	t-value	p-value
Age	46 ± 5.720	43.87 ± 5.489	-5.7	0.306
Height (cm)	167.87 ± 9.01	167.34 ± 7.92	0.351	0.866
Weight (kg)	73.53 ± 10.06	73.27 ± 9.63	0.396	0.941
BMI	25.97 ± 1.46	26.04 ± 1.28	-0.769	0.895

Table 2 shows the comparison between the experiment and the CPT group in pre-intervention stroke measuring tools among the chronic stroke population. There is no statistically significant difference between the experiment and the CPT group in the BBS with p > 0.05 and TCT with p > 0.05 in pre-intervention.

**Table 2:** Intergroup comparison between control and experimental groups of BBS and TCT values on day one BBS, Berg Balance Scale; CPT, conventional physiotherapy; TCT, Trunk Control Test

Outcome variables	CPT group (mean ± SD)	Experiment group (mean ± SD)	t-value	p-value
BBS	37 ± 2.976	34.33 ± 4.337	1.964	0.06
TCT	50.27 ± 16.624	48.33 ± 10.567	0.38	0.707

Table 3 shows within group analysis of balance and trunk control for both groups. The CPT group shows significant improvement in the Berg Balance score (p = 0.001, t = 14.642). The TCT value in the CPT group shows significant improvement after a four-week intervention (p = 0.001, t = 8.439). Similarly, experimental groups show significant improvement with p = 0.001 and t = 21.236. The TCT in the experimental group shows significant improvement with p = 0.001 and t = 12.508.

**Table 3:** Depiction of within group analysis of BBS and TCT pre-intervention and post-four-week intervention * significant i.e., p < 0.05 BBS, Berg Balance Scale; CPT, conventional physiotherapy; TCT, Trunk Control Test

Groups	Variables	Pre-test (mean ± SD)	Post-test (mean ± SD)	t-value	p-value
						CPT	BBS	37 ± 2.976	41.67 ± 3.063	14.642	0.001*
TCT	50.27 ± 16.624	73.20 ± 11.334	8.439	0.001*		
Experimental	BBS	34.33 ± 4.337	43.80 ±4.663	21.236	0.001*
	TCT	48.33 ± 10.567	78.33 ± 11.697	12.508	0.001*

Table 4 shows the group analysis of the BBS and the TCT between the CPT and experiment groups. There is a statistically significant difference between the experiment and the CPT group in BBS with p = 0.001 and t = 8.759, although the TCT score was insignificant with p = 0.061 and t = 1.950.

**Table 4:** Comparison between experiment and control group in post-pre-test intervention stroke measuring tools among the chronic stroke population * significant i.e., p < 0.05 BBS, Berg Balance Scale; CPT, conventional physiotherapy; TCT, Trunk Control Test

Outcome variables	CPT group (mean ± SD)	Experiment group (mean ± SD)	t-value	p-value
BBS	4.67 ± 1.234	9.47 ± 1.727	8.759	0.001*
TCT	22.93 ± 10.525	30 ± 9.289	1.95	0.061

## Discussion

In this study, we aim to analyze the effects of proprioceptive training on balance and trunk control among individuals with a chronic stroke.

Balance

The findings of the CPT group show a significant (t = 14.642, p = 0.001) improvement in the BBS. Strength training given to the CPT group might have resulted in modifications in neuromuscular factors that eventually helped in improving motor control, coordination, and proprioceptive feedback. Modifications in neuromuscular factors such as greater motor unit recruitment, improved synchronization of muscle activation, and increased muscle fiber size and quality can aid in retraining the brain’s motor circuits, allowing stroke patients to restore balance. Similar findings were also observed by Jeon and Hwang in a study done on 20 hemorrhagic as well as ischemic stroke subjects. They found that bilateral lower limb strength training improved the balance [23]. Evidence suggests that the physiological integrity of the corticospinal tract impacts motor function in the lower limb in stroke patients [24]. It is clearly recognized that certain fibers of the corticospinal tract do not cross at the pyramidal decussation. The expected proportion of uncrossed tracts is between 10% and 20%. These uncrossed ipsilateral tracts have been suggested as a potential post-stroke healing mechanism, and researchers claim that bilateral strength training might activate them [23].

When comparing pre-test and post-test scores, the findings of the BBS in the experimental group showed a significant (p < 0.05) improvement in balance. Proprioceptive training, including weight shifting, body rotations, and demanding postures, can all aid in enhancing balance. The exercises included in the proprioceptive training group might have influenced neuronal circuits; the active involvement of participants results in the excitation of the higher center, causing the brain to adapt and restructure itself [25]. In addition to neuromuscular factors, these excited neuronal pathways may lead to better motor control and balance. The findings of this study are consistent with a previous study done by Lobo et al. on 30 hemorrhagic as well as ischemic acute stroke subjects; they observed improvements in weight-wearing symmetry directing the center of gravity to the midline. The possible reason could be that compelled weight-bearing on the affected limb facilitates baroreceptors’ feedback to the higher center, resulting in improved joint stability, postural control, and balance [26]. Another meta-analysis of 16 studies evaluated the effects of sensory retraining of the lower limb on balance after stroke and found significant improvement in balance [27]. This proprioceptive training intervention appears to be an effective method for addressing imbalances in chronic stroke patients.

A significant difference (t = 8.759, p = 0.001*) in BBS score was observed between the two groups when compared after four weeks of intervention. This result is similar to the studies done earlier, e.g., a study done in 2017 by Chae et al. on the chronic stroke population, which shows significant improvement in balance. A potential reason behind this could be that proprioceptive training, when given along with CPT, helped in recruiting additional neuronal pathways and activating muscles [28]. These changes resulted in successful assessment outcomes. It is conceivable that the chosen therapy effectively addressed the unique needs of the chronic stroke population.

Trunk control

Within-group comparison of TCT scores in the CPT group shows significant (p = 0.001, t = 8.439) improvement. The exercises included in the CPT group might have involved a lengthy lever arm, and the upper body muscles had the capacity to provide enough torque to load the trunk muscles. This indirect loading of trunk muscles could have helped to recruit more muscle fibers. This new muscle fiber recruitment helped to improve strength and trunk control. In a study done by Lee et al. on 30 subjects, they found that lifting of the upper extremity activates the trunk muscles [29,30].

Within-group comparison of TCT scores in the experimental group also shows significant improvement (p = 0.001, t = 12.508). Stroke survivors have poor trunk position sense, which can lead to trunk instability. By engaging in activities that challenge balance, weight shifting, and coordination, such exercises appear to influence proprioceptive pathways. These simulations encourage the transfer of sensory information to the brain, promoting the reorganization and rebuilding of neuronal connections. It contributes to the strengthening of neuronal connections between the sensory and motor areas of the brain and provides greater movement coordination and trunk control. Our findings correspond to research done by Jung et al. in 2014 on 18 hemiparetic chronic stroke subjects, who found that weight shifting helps to improve trunk control in chronic stroke patients [31]. Another meta-analysis done by Apriliyasari et al. in 2022, which included 17 clinical trials involving 447 subjects, had similar findings to ours, i.e., that proprioceptive training is effective in improving trunk control [32].

However, there were no significant differences found in trunk control between CPT and the experimental group post-four-week intervention; the proprioceptive and strength training exercises included in the experimental group might not be able to trigger the trunk muscles sufficiently. A longer treatment duration and a larger sample size may help us find more inside. The negligible results seen might be attributable to random fluctuation or chance rather than a true absence of differences between the groups. Larger research with more people might yield more clear results.

While our study has yielded valuable insights, it is essential to acknowledge some limitations that provide opportunities for further investigation and improvement. The study’s small sample size allowed for in-depth analysis and served as a preliminary exploration of the subject, offering valuable initial findings. Although patients were not blinded to their assigned groups, this openness fostered a supportive environment and allowed for meaningful patient engagement. The prominence of ischemic stroke subjects in the study has provided valuable stroke type-specific information, enabling us to better understand this specific population. While the findings may be more directly applicable to individuals with similar characteristics, they offer valuable insights that can inform tailored approaches for different groups. Although time restrictions limited follow-up capacity, the study provided essential short-term observations, prompting the need for future long-term investigations to fully explore the potential benefits. Overall, this study acts as a stepping stone for further research, building upon its strengths to enhance the understanding of the subject matter.

## Conclusions

These results show that between-group comparison after the four-week intervention has significant improvement in balance but no significant improvement in trunk control in this specific group of participants. However, significant improvement has been seen within the groups. It is likely that an extended intervention time or a different form of intervention may be required to achieve substantial gains in these areas. Future research might look at other outcome measures or the impacts of other types of therapies to see which ones are most helpful at increasing balance, trunk control, and functional reach.
